# AUF1 and HuR: possible implications of mRNA stability in thyroid function and disorders

**DOI:** 10.1186/1756-6614-4-S1-S5

**Published:** 2011-08-03

**Authors:** Bogusz Trojanowicz, Henning Dralle, Cuong Hoang-Vu

**Affiliations:** 1 Universitätsklinik und Poliklinik für Allgemein-, Viszeral- und Gefäßchirurgie, Martin-Luther Universität, Halle

## Abstract

**Abstract:**

RNA-binding proteins may regulate every aspect of RNA metabolism, including pre-mRNA splicing, mRNA trafficking, stability and translation of many genes. The dynamic association of these proteins with RNA defines the lifetime, cellular localization, processing and the rate at which a specific mRNA is translated. One of the pathways involved in regulating of mRNA stability is mediated by adenylate uridylate-rich element (ARE) binding proteins. These proteins are involved in processes of apoptosis, tumorigenesis and development. Out of many ARE-binding proteins, two of them AUF1 and HuR were studied most extensively and reported to regulate the mRNA stability *in vivo*. Our previously published data demonstrate that both proteins are involved in thyroid carcinogenesis. Several other reports postulate that mRNA binding proteins may participate in thyroid hormone actions. However, until now, exacts mechanisms and the possible role of post-transcriptional regulation and especially the role of AUF1 and HuR in those processes remain not fully understood. In this study we shortly review the possible function of both proteins in relation to development and various physiological and pathophysiological processes, including thyroid function and disorders.

## Introduction

Cytoplasmic stability of eukaryotic mRNA is a substantially important check point in the control for gene expression. During differentiation of the cell some mRNAs are expressed stronger and some others are degraded or down regulated. Such a post-transcriptional gene-inactivation (gene silencing) occurs through alterations in translational efficiency and in mRNA stability. Stability of mRNA is mainly controlled by RNA interference processes (e.g. siRNA, microRNA) and RNA binding proteins, which act to selectively degrade or stabilize mRNAs. In mammals, a common feature of many unstable mRNAs is the presence of an Adenylate-Uridylate-rich element (ARE) within the 3'-untranslated region (3'-UTR) [1-3]. This ARE in the 3'-untranslated region is a part of the regulation system, which is responsible for the mRNA degradation or stabilisation and is linked to an interaction with ARE binding proteins. Out of many ARE-binding proteins studied, two of them AUF1 and HuR were investigated most extensively and demonstrated to affect the mRNA stability *in vivo*. AUF1 (A+U rich RNA-binding factor 1, heterogeneous nuclear ribonucleoprotein D) is expressed as a family of four proteins designated by their molecular masses as p37, p40, p42 and p45 arising from a single transcript. HuR (human antigen R) is a ubiquitously expressed member of the embryonic lethal abnormal vision (ELAV) family [4]. Although there are some examples demonstrating stabilizing function of AUF1, it is generally related to degradation of target mRNAs while HuR is known to promote stabilization of several transcripts by enhancing their stability, altering their translation, or performing both functions [5-7]. Both proteins are mainly expressed in nucleus but their function is related with cytoplasmic localisation. Predominantly nuclear AUF1 and HuR shuttle between the nucleus and cytoplasm via nucleocytoplasmic shuttling sequences called DNS and HNS, respectively. In case of AUF1, its nuclear import is mediated by transportin-1 [8,9]. The mechanisms of AUF1 and HuR are believed to be mediated by binding-competition to target mRNAs. It is postulated that AUF1 and HuR could functionally act with target mRNAs both within the nucleus and in the cytoplasm [10]. It suggests that precise mechanism of antagonising and/or cooperative actions of AUF1 and HuR is not entirely understood. Both proteins do not posses any enzymatic activity, but were identified with ARE-mRNA-protein-ligand complexes. Previous reports identified the ubiquitin-conjugating enzyme E2I and three RNA binding proteins: NESP-1, NSAP-1 and IMP-2, as AUF1 interacting proteins. Importantly, NSEP-1 revealed an endoribonuclease activity, providing possible explanation for AUF1-mediated poly(A) shortening [11]. With regard to HuR, affinity chromatography and co-immunoprecipitation experiments identified four protein ligands SETa, SETb, pp32 and acidic protein rich in leucine (APRIL) [12]. First three had been previously characterised as inhibitors of protein phosphatase 2A (PP2A) regulating its ability to convert between holoenzyme forms upon stimulation [13,14]. PP2A is known serine/threonine phosphatase participating in diverse cellular processes such as proliferation, cell cycle, DNA replication, development and morphogenesis [15]. Recently, HuR and AUF1 were identified as components of the RNA-induced silencing complex (RISC). In this study HuR employed AUF1 as cofactor to promote p16 mRNA decay [16].

ARE-elements are found in many mRNAs encoding proteins related to normal and neoplastic cell growth and must be considered as a crucial target element in mammalian cells. The human genome project revealed that many mRNAs encoding proto-oncogenes (i.e. c-myc, c-jun, c-fos), growth factors such (i.e. EGF, VEGF), cytokines (i.e. TNFα) and cell cycle regulators (i.e. cyclin A, B1, D1, p21, p27) contain ARE-motifs [17,18]. Analysis of thyroid related genes revealed that many of them contain these motifs and may be regulated at the post-transcriptional level. These include mRNAs related with thyroid function (pendrin, NIS, MCT8, peroxiredoxins, TPO, dual oxidase 1), regulation of thyroid function (TSHR), response to thyroid hormones (selenoprotein P, THR alpha and beta, THR associated protein 6, D1), thyroid development (Nkx2-1, PAX8, HHEX, EYA1, Hox-A3, Hox-A5, PAX9) and thyroid pathology (thyroid adenoma associated protein, papillary thyroid carcinoma encoded protein, thyroid cancer protein) (Table 1). However whether these mRNAs may serve as potential targets for AUF1 and HuR is still unclear. It is worth to note that both proteins may also interact with some mRNAs by employing non-canonical ARE-sites [19,20].

**Table 1:** ARE-containing mRNAs related to thyroid function, development and disorders. According to AREsite http://rna.tbi.univie.ac.at/cgi-bin/AREsite.cgi.

	ARE-motifs				
mRNA	AUUUA	WTAUUUATW	WWWWAUUUAWWWW	WWAUUUAWW	Others

Selenoproten P	6	1	1	2	
MCT8	5				
ID2	3	1	1	1	1
Pendrin	6	2	1	2	4
THR alpha	2			1	
THR beta	14	1		2	
Thyroid adenoma associated protein	18	1	2	6	6
TTF-1 (Nkx2-1)	6	3	2	4	4
TSH-R	2				
TPO	3			2	
THR associated protein 6	2			1	
Papillary thyroid carcinoma encoded protein	10	2	1	3	2
Dual oxidase 1	1				
Thyroid cancer protein	5	1	1	2	2
PAX8	3	1		2	3
NIS	1				
D1	3				
Homebox protein HEX (HHEX)	2				
Eyes absent homolog EYA1	5			2	1
Homebox protein Hox-A3	7	1	1	1	3
Homebox protein Hox-A5	1	1		1	
PAX9	4	1		3	
Peroxiredoxin 6 (PRDX6)	2				
Peroxiredoxin 3 (PRDX3)	1				
Peroxiredoxin 4 (PRDX4)	1				

## Developmental expression of AUF1 and HuR

Both proteins are synchronous, but differential expressed during murine development. It had been demonstrated that there is no major translation control of AUF1 and HuR production, but their expression varies noticeably within tissues and at differential developmental stages. The tissue distribution of both proteins demonstrated that yolk-sack and fetal liver possessed very weak levels of AUF1 and HuR, contrary to strong expression in tail, limb bud, brain and heart between E13.5 and E16.5. Adult spleen, thymus, testis and intestine were very abundant in AUF1 and HuR, contrary to brain, liver, heart, kidneys, muscle, lungs and stomach, demonstrating low or no expression of both proteins. AUF1 and HuR revealed a coincident spatio-temporal tissue distribution from 10-somite stage to late embryogenesis [21,22].

The data coming from human tissues revealed that AUF1 and HuR are also strictly involved in liver de-differentiation, development and progression of hepatocellular carcinoma. Those processes are tightly regulated by MAT1A (methionine adenosyltransferase 1A)- and MAT2A (methionine adenosyltransferase 2A. Both enzymes control the levels of S-adenosylmethionine (SAM), the key regulator of hepatocytes proliferation and differentiation. MAT1A is expressed in adult liver, whereas MAT2A is detected extrahepatic and is related with liver proliferation. The analysis of those expression patterns revealed that AUF1 and HuR are crucial post-transcriptional regulators of MAT1A and MAT2A mRNA stability. The authors demonstrated that AUF1 and HuR levels were upregulated in a time-dependent manner during liver de-dedifferentiation and the function of HuR is regulated by its methylation status. The interaction of HuR with MAT2A mRNA in the presence of SAM led unexpectedly to MAT2A mRNA decay, whereas its absence promoted HuR-mediated accumulation of MAT2A mRNA. It is worth to note that addition of SAM reduced the AUF1 interaction with this mRNA. The analysis of rat liver development revealed that there is a switch in the expression from MAT2A to MAT1A during embryogenesis. Proliferating liver express increased levels of SAM-methylated HuR which leads to decreased MAT2A mRNA expression and elevated levels of MAT1A mRNA. Furthermore, increased levels of AUF1 and HuR, and decreased levels of methylated-HuR were detected in hepatocellular carcinoma tissues. Normal liver tissues express very low levels of AUF1 and HuR, and high levels of methylated-HuR [23].

*In vivo* data on AUF1 and HuR knock-out mice models demonstrated that both proteins may play an important role in the development. Investigations of AUF1 -/- mice revealed symptoms which share similarity with human atopic dermatitis such as pruritus, chronic dermatitis, cutaneous atopy, xerosis, elevated serum IgE levels and staphylococcal skin infection [24]. Cunningham et al. observed the presence of thyroid disorders in patients suffering from dermatitis herpetiformis. However the association between the clinical or serological thyroid abnormalities and skin disease could not be explained [25]. Recently different skin findings and accompanying dermatoses could be detected in patients with thyroid diseases. The presence of each dermatosis, chronic urticaria, vitiligo and pruritus were found to be significantly higher in the patient group with thyroid disorders than in the control group. Autoimmune-hyperthyroidism patients demonstrated significantly higher incidence of vitiligo and diffuse alopecia, while vitiligo alone was found to be significantly higher in autoimmune hypothyroidism patients than in the control group [26]. It had been demonstrated that AUF1-deficient mice revealed abnormal stabilisation of TNFα and IL-1β mRNAs. Such an excessive proinflammatory cytokines production resulted in endotoxic shock, loss of renal and liver function and in end effect caused increased mortality [27]. In studies by Sadri et al. AUF1-deficiency led to disturbance in mature splenic B cell populations and impaired humoral immune responses. The authors observed decreased number of splenic lymphocytes, increased presentation of immature marginal zone lymphocytes and reduced number of mature follicular B lymphocytes [28].

More severe abnormalities were observed in HuR -/- animals. It had been demonstrated that HuR was crucial for midgestational embryonic development. HuR expression increased in murine placenta between E12.5 and E13.5. Immunohistochemical analysis revealed its presence in both embryonic and extraembryonic layers; however HuR nucleo-cytoplasmic localisation varied between maternal and fetal sides. It was dominantly nuclear in maternal decidua cells and embryo-derived trophoblasts giant cells and spongiotrophoblasts. Contrary, labyrinth-trophoblasts showed nuclear and strong cytoplasmic staining. The presence of HuR in cytoplasm at this stage was probably related with its function. Furthermore, the HuR -/- led to midgestational death. In the absence of HuR the labyrinth branching morphogenesis and syncytiotrophoblast differentiation were impaired and caused not sufficient vascularization, labyrinthine apoptosis, and in end effect insufficient nutrient transport and embryos death. Most of the embryos died between E17.5 and E19.5. They revealed reduced size of skeleton and limbs and decreased spleen size. Furthermore, the lungs of HuR -/- mice revealed additional dysmorphologies, whereas the stomach and pancreas were properly located. However, whether the absence of HuR could lead to thyroid abnormalities was not investigated. It is worth to note that HuR was able to interact with several transcription factors crucial not only for proper morphogenesis, patterning and specification, but also for thyroid development such as Hox-A5 [29].The absence of Hox-A5 is related to disorganized follicle formation; decreased TPO; Nkx2.1, Titf2, altered Pax8 gene expression, but normal serum T4 level. As demonstrated in Table 1, these mRNAs bear ARE-sequences and are crucial for thyroid development (Table 2). However, whether their mRNA stability is regulated in ARE-dependent manner, especially in participation of AUF1 and HuR, is not clear.

**Table 2:** Thyroid developmental markers containing ARE-sequences and phenotypes studied on double knock-out mice, modified from [85].

Double knock-out	Thyroid phenotype
Pax8 -/-	Regression of thyroid primordium at E11.5 Thyroid precursor cells disappear at E12 No follicles, no Tg, no TPO; thyroids exclusively consist of C-cells (Ttif1-positive)

Pax9 -/-	Lack of ultimobranchial body formation

Ttif1/Nkx2.1 -/-	Thyroid precursor cells disappear at E10.5-11.5; Mice lack C-cells and thyroid follicular cells

Hhex -/-	Thyroid anlage present at E9; Absence of thyroid primordium at E10

Eya1 -/-	lack of fusion between ultimobranchial bodies and thyroid lobes; thyroid hypoplasia; severe reduction in number of C-cells and follicular cells;

Hoxa3 -/-	Thyroid hypoplasia; persistent ultimobranchial bodies with C-cells

Hoxa5 -/-	Disorganized follicle formation; decreased TPO; Nkx2.1, Titf2, Pax8 gene expression altered, but normal serum T4 level

## Thyroid hormone and TSH-mRNA

There is still a growing body of evidence that actions of thyroid hormone (TH), thyroid function and disorders may also be regulated at post-transcriptional level by mRNA binding proteins. Krane at al. demonstrated that TH is able to regulate the stability of thyroid stimulating hormone (TSH) subunit mRNA. They showed that half-life of the TSH beta mRNA from rat pituitary cultures treated with triiodothyronine (T3) was shorter than that observed in corresponding controls. Furthermore, TSH mRNA was destabilized due to the loss of a portion of the poly(A) tail upon TH treatment [30]. Staton at al. postulated that such a mechanism is highly conserved across the species. In addition to previous studies they observed that simple deadenylation of TSH messenger body is insufficient to induce its decay [31]. This suggests the triggering of additional mechanism such as exonuclease digestion and actions of other still not identified mRNA binding proteins. Recently, Goulart-Silva et al. demonstrated a rapid effect of T3 on TSHβ mRNA stability. They showed that surgically hypothyroided rats revealed an increased TSHβ transcript poly(A) tail, its elevated content in ribosomes and enhanced attachment to cytoskeleton. Furthermore, hypothyroid rats subjected to acute T3 treatment demonstrated a reduction of TSHβ mRNA poly(A) tail and its weakened recruitment to ribosomes. Euthyroid rats did not present any changes in TSHβ mRNA stability, but revealed increased binding of TSHβ transcripts to ribosomes [32]. Although exact mechanisms or proteins participating in mRNA degradation are not fully identified, Leedman et al. isolated a cytoplasmic trans-acting factor (80-85 kDa) capable to bind the 3’-UTR of TSHβ transcripts. Furthermore, its binding to TSHβ transcripts was positively regulated by T3 [33]. With regard to AUF1 and HuR, their actions in regulating the TSHβ transcript stability may be elusive at present, but it is worth to note that AUF1 function is associated with polysomes [10].

## Thyroid hormone and other mRNAs

Murphy et al. observed that growth hormone (GH) mRNA from TH-depleted rats is less abundant than euthyroid controls, but its stability is elevated due to the increased length of poly (A) tail. The authors observed that modulation of GH poly (A) is related to nucleus and its length may be regulated due to the interactions with free ribonucleoproteins. The existence of such a nuclear mechanism resulting in cytoplasmic stabilisation of mature GH mRNA raises the question which mRNA binding proteins may mediate these effects [34]. Recent studies by Goulart da Silva et al., revealed that TH acutely increases GH mRNA translation rate and GH secretion in hypothyroid rats. It had been shown that soon after T3 administration, increased GH labelling was observed in the perinuclear region of somatotrophs and that these events coincided with cytoskeleton rearrangements. Indeed, the authors identified GH mRNA in complexes with F-actin, which provided additional protection for GH mRNA transcripts. Furthermore, as the authors postulated, insufficient GH synthesis in hypothyroid specimens resulted not only from reduced GH transcription rate but also decreased GH mRNA half-life [35]. This novel finding directing GH mRNA soon after T3 administration to polysomes is related with TH nongenomic actions. However, whether additional molecular mechanisms are involved in those processes, remains an open question.

With regard to other mRNAs, the effects of TH on cardiovascular development may also be mediated by actions of mRNA-binding proteins. Minamisawa et al. demonstrated that stability of sarcolipin mRNA, an atrium-specific sarcoplasmic reticulum protein, is affected by thyroid hormone actions. Analysis of atria obtained from hyperthyroidism-induced and T3-treated neonatal rat atrial myocytes revealed accelerated decay of sarcolipin mRNA. The hyperthyroid mice revealed cardiac hypertrophy and sinus tachycardia. Contrary, hypothyroidism did not result in any changes of cardiac morphology and sarcolipin mRNA stability [36]. It had been demonstrated that myocardial relaxation and associated cardiac hypertrophy and heart failure may be related with AUF1-mediated decrease of sarco(endo)plasmic reticulum calcium ATPase 2a (SERCA2a) mRNA stability. In those studies AUF1 was identified as a critical factor of these events. Interaction of AUF1 with SERCA2a 3' UTR was identified predominantly in nucleus, suggesting that AUF1-mediated decay of SERCA2a mRNA starts within the nucleus and further continues during shuttling to the cytoplasm [37].

The further role of AUF1 was shown in studies concerning the mRNA stability of Kv4 channels. Expression of cardiac myocyte Kv4 channels is down-regulated in hypertrophy and leads to decrease in the transient outward current. Studies in vitro demonstrated that employment of angiotensin II may recapitulate these effects and is accompanied by up-regulation of AUF1, which in turn binds and destabilises Kv4 mRNA [38].

Studies on cultured rat brown adipocytes revealed that at higher doses T3 inhibited deiodinase II (D2) activity. Similar effects were induced after administration of triiodothyroacetic acid (Triac) , a natural thermogenic compound in brown adipocytes. Low concentrations of Triac increased D2 activity, while its higher doses inhibited D2 activity. The authors postulate that D2-inhibiton by employing high T3 doses may exist as a regulatory pathway in which D2 triggers down-regulation of T3, against its excess. D2 activity is inhibited in hyperthyroidism, and decreases further after chronic cold exposition. This suggests that high T3 concentrations in brown adipocytes switch off the mechanisms of T3-production via D2. Furthermore, the inhibition of D2 activity was found on post-transcriptional level and was related with proteasome actions. It had been demonstrated that addition of ubiquitin led to D2 inactivation whereas protein deubiquitintating enzyme-1 reversed ubiquitin-mediated inactivation and prolonged D2 half-life. Furthermore, D2 was also stabilised by employing of proteasome inhibitor. Deubiquitintating enzyme-1 was found noticeably increased in brown adipocyte tissue upon cold exposure and was associated with increased D2 stability.

Although there is no direct evidence that T3 regulates proteasome activity or increase D2 decay, the authors could demonstrate that T3 at high doses and THR beta participate in regulation of D2 mRNA stability, T3 by shortening D2 half-life and induction of D2-degradation, and THR beta by stabilisation of D2 activity [39-43].

TH is also implicated in regulation of neuroserpin mRNA stability. Neuroserpin is a serine protease inhibitor of the serpin family playing an important role in neural development and adult neural function. Neuroserpin dysfunction is related with different neurodegenerative diseases. It has been shown that its 3’UTR contains ARE motifs and interacts with HuD protein, belonging as HuR to the ELAV family. HuD is able to bind neuroserpin mRNA and its overexpression was associated with increased mRNA stability. Employment of T3 led to decreased half-life of neuroserpin, probably due to the HuD repression. The authors also suggest the involvement of other mRNA binding proteins [44].

In studies with retinoic acid receptors (RAR) it has been demonstrated that administration of TH led to enhanced mRNA levels of RARβ, decreased RXRγ and had no influence on RXRα. In addition to positive transcriptional regulation of RARβ by TH, investigations concerning the regulation of RXRγ, revealed that TH regulate its mRNA stability post-transcriptional [45]. As RXRγ mRNA bears the ARE-motifs, it is a possible partner for ARE-binding proteins.

## Trace elements

Recent studies revealed that also trace elements such as selenium, calcium or iron may regulate the mRNA stability of proteins related to their transport or metabolism [46-49]. Studies *in vitro* revealed potential roles of calcium and phosphate in AUF1-mediated mRNA stability of parathyroid hormone (PTH). PTH mRNA levels are post-transcriptional increased by hypocalcemia and decreased by hypophosphatemia. AUF1 was identified as a critical factor protecting and stabilizing PTH transcripts [50]. With regard to thyroid function, it has been demonstrated that iodide and selenium may affect the stability of thyroid-related mRNAs. Bermano et al. showed that selenium depletion decreased the stability of cytosolic glutathione peroxidase mRNA. Furthermore, the incorporation of Se-cysteine at specific UGA codons in the selenoenzyme mRNAs was dependent on the presence of stem-loop structures in the 3'-UTRs of these mRNAs. It was demonstrated for selenoenzymes type I (D1) and type III deiodinases (D3) revealing that their translation efficiency was dependent on the structure or sequence of the 3'- UTR [51]. The existence of Se-dependent post-transcriptional mechanisms regulating D1 mRNA stability was also reported in study by Riese et al., suggesting regulation in a sex and tissue specific manner [52]. Although involvement of AUF1 and/or HuR in Se-regulated mRNA stability may be elusive at present, its worth to note that 3'- UTR of D1 bears ARE-motifs and may be regulated in ARE-dependent fashion.

Recent studies demonstrated that acute iodide administration decreased the mRNA stability of sodium-iodide symporter (NIS). The authors observed that NIS transcripts and the length of NIS poly (A) tail were significantly reduced during all periods of iodide treatment. The authors postulate that this mechanism involves the interaction of trace elements such as iodide with proteins bound to untranslated regions of mRNAs. This novel mechanism provide a new insight by which iodide may exert its autoregulatory function on thyroid gland [53]. However whether iodide or other trace elements may bind and regulate the activity of exosome and/or proteasome components in ARE-dependent manner remains to be identified.

## Thyroid cancer

Current data from our group revealed that AUF1 and HuR may be involved in thyroid pathology by regulating the stability of several factors related to cell cycle and proliferation. We demonstrated that thyroid cancer tissues expressed AUF1 significantly stronger than benign tissues. Furthermore, AUF1-knock-down in follicular thyroid carcinoma cells FTC-133 led to decreased proliferation rates accompanied by increased expression of cell cycle inhibitors and down-regulation of cell cycle promoters. The analysis of AUF1-depleted clones revealed that in addition to AUF1-down-regulation, all clones demonstrated also decreased HuR levels [54]. However exact mechanisms, thyroid-specific partners for AUF1 and HuR, the influence of AUF1 and HuR on thyroid specific functions and genes, the role of mRNA stability in thyroid carcinogenesis are still not fully understood. Recent studies by Braun et al. identified altered microRNA signatures as potent post-transcriptional markers for undifferentiated thyroid carcinoma that promote its de-/transdifferentiation and invasion [55].

**Figure 1:**
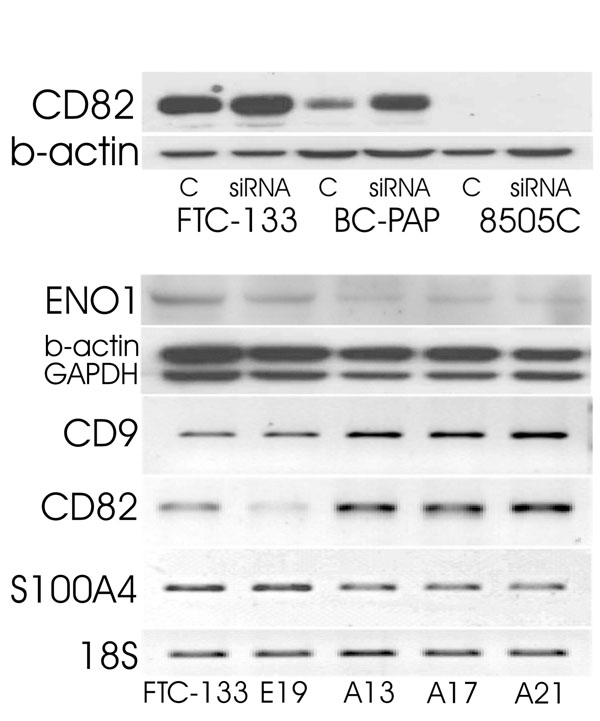
Upper panel: expression of CD82 in thyroid carcinoma cell lines treated with siRNA targeting AUF1. Lower panel: expression of CD9, CD82, GAPDH, ENO1 and S100A4 in wild type cells FTC-133, FTC-133-EGFP control cells (E19) and FTC-133 transfectants expressing AUF1 targeting shRNAs (A13, A17, A21). Increased levels of tumor suppressors (CD9 and CD82) and subsequent decrease of tumor promoters (ENO1, GAPDH and S100A4) are visible. Note that 8505C control and AUF1-siRNA cells are CD82 negative. 18S and B-actin served as normalizing markers.

Our previously published data revealed that expression of several tumor suppressor genes (TSG) and tumor promoting factors, may serve as novel biomarkers for thyroid carcinoma. We demonstrated that down-regulation of CD9, CD82 and RKIP may reflect an increased *in vivo* metastatic potential of thyroid cancer cells [56,57]. Inversely, increased expression of S100A4 and ENO1 was correlated with increased proliferation and metastatic potential of thyroid carcinoma [58]. Investigations performed on AUF1-depleted thyroid cancer cell lines revealed a cross-link between AUF1 and CD9, CD82, S100A4 and ENO1. We demonstrated that AUF1 knock-down led to increased expression of two tumor suppressor genes CD9 and CD82. Subsequently, decreased expression of tumor promoting S100A4, GAPDH and ENO1 was observed (Fig. 1).

**Figure 2:**
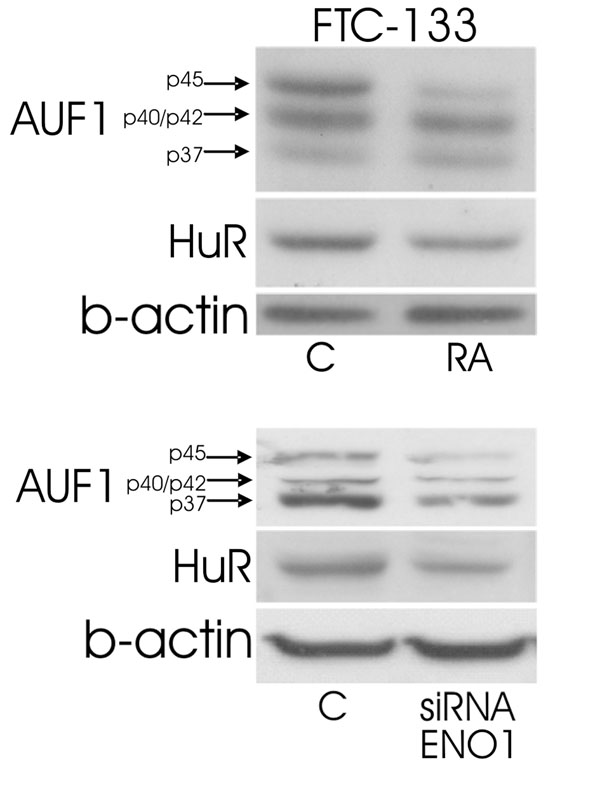
Expression of AUF1 and HuR after RA pre-treatment and ENO1-siRNA employment. Upper panel: FTC-133 cells pre-treated with RA revealed decreased levels of AUF1 and HuR. Lower panel: ENO1 knock-down led to decreased levels of AUF1 and HuR. B-actin served as normalizing marker.

It had been demonstrated that retinoic acid (RA) treatment induced beneficial effects in therapy of solid cancers [59,60] including thyroid carcinomas [61-63]. Cell culture experiments performed on thyroid carcinoma cell lines revealed that RA treatment affects thyroid-specific functions, cell-cell or cell-matrix interaction, differentiation markers, growth, and tumorigenicity [64]. Administration of RA demonstrated an anti-proliferative effect on the follicular thyroid carcinoma cell lines FTC-133 and FTC-238. Furthermore, pre-treatment of these cell lines with RA resulted in decreased in-vitro proliferation rates and reduced tumor cell growth of xenotransplants [65]. Our published data revealed that RA led to significantly decreased levels of of glyceraldehyde-3-phpsphate dehydrogenase (GAPDH), pyruvate kinase isoenzymes M1/M2 (PKM1/M2), peptidyl-prolyl cis-trans isomerase A (PPIA), transketolase (TKT), annexin A2 (ANXA2), glutathione S-transferase P (GSTP1) and peroxiredoxin 2 (PRDX2) as compared to untreated control. Investigations on thyroid tissues demonstrated that the same proteins were significantly up-regulated in follicular, papillary and undifferentiated thyroid carcinomas [66]. In addition to previously published data we found that RA pre-treatment led to down-regulation of AUF1 and HuR. Also depletion of ENO1 led to decreased levels of AUF1 and HuR (Fig. 2).

## Other types of cancer

Both proteins were reported to be also related with other malignancies, and like in case of thyroid cancer, were expressed synchronous. Given that both proteins bind to common target mRNAs such an expression pattern is partly explainable. In studies on transgenic mice the overexpression of AUF1 led to development of sarcomas with strong expression of cyclin D1 [67]. Other *in vivo* mice models demonstrated that malignant lung tissues expressed AUF1 significantly stronger as compared with normal and benign lung tissues. The same expression pattern was reported for HuR protein [68]. With regard to HuR expression, its increased levels were demonstrated for malignant tissues of tongue, gingival, colon, breast, ovary and stomach as compared to corresponding controls [69,70].

It had been shown that approximately 80% of anaplastic lymphoma kinase (ALK)-positive lymphomas express the fusion protein called nucleophosmin-anaplastic lymphoma kinase (NPM-ALK), which constitutively activates ALK tyrosine kinase and causes abnormal induction of down-stream signaling resulting in malignant transformation. Studies *in vitro* revealed that AUF1 was co-localised with NPM-ALK in the same cytoplasmic loci. Subsequent hyperphosphorylation of AUF1 in NPM-ALK expressing cells was also observed. It is worth to note that AUF1 hyperphosphorylation was associated with elevated stability of several target mRNAs encoding proteins crucial for cell proliferation and cell survival such as c-myc, and cyclin D1, cyclin A2, cyclin B1, and cyclin D3 [71].

Overexpression of AUF1 (especially isoforms p37 and p45) resulted in enhanced translation of internal ribosome entry site (IRES) of Hepatitis C virus (HCV), which is one of the major agents causing a virus-related hepatitis, liver cirrhosis and hepatocellular carcinoma. Depletion of AUF1 significantly hampered infection by HCV [72]. Similar mechanisms were also reported by depletion of insulin-like growth factor 2 mRNA binding protein IGF2BP1 [73].

Increased expression of AUF1, heterogeneous nuclear ribonucleoprotein K (HNRNPK), MutS homolog 2 (MSH2) and grainyhead-like 2 (GRHL2), and subsequently elevated activity of human telomerase reverse transcriptase (hTERT) were detected in human oral squamous cell carcinoma cells (OSCC) when compared to normal cells, which do not exhibit hTERT activity. RNAi mediated knock-down of AUF1, MSH2 and GRHL2 resulted in decreased proliferation rates and hTERT promoter activity. Depletion of HNRNPK inhibited only proliferation of the cells without affecting the hTERT promoter activity [74].

Prostaglandin A2 (PGA2) is an experimental anti-cancer agent associated with decreased levels of cyclin D1 and decreased proliferation of cancer cells. It has been demonstrated that employment of PGA2 induced AUF1 expression and resulted in degradation of cyclin D1 mRNA in non small cell lung cancer (H1299) and breast carcinoma (MCF-7) cells. The other breast carcinoma cell line tested MDA-MB-453, bearing a large deletion in cyclin's D1 3'UTR, responded with unaltered cyclin D1 mRNA upon PGA2 treatment [75]. In melanoma cells increased elevated levels of interleukin 10 (IL-10) resulted in decreased cytosolic AUF1 levels as compared with normal melanocytes [76].

## Non-cancerous actions and disorders

The role and actions of AUF1 and HuR were also reported in other non-cancerous disorders. AUF1 proteins were identified as novel autoantigens in systemic lupus erythematosus (SLE) and other associated autoimmune rheumatic disorders. Autoantibodies to AUF1 were detected in 33% of SLE patients, 20% of patients with rheumatioid arthritis, 17% of patients with mixed connective tissue disorders and below 10% of patients with other related rheumatic diseases. Healthy controls were negative for AUF1 autoantibodies [77]. The presence and the role of HuR autoantibodies are discussed in non-neuronal tissues [78].

The role of post-transcriptional gene regulation was also studied in the cells upon low oxygen levels. It has been demonstrated that in hypoxia conditions, HuR up-regulates the expression of two major hypoxia-inducible proteins, VEGF and HIF-1α. Furthermore, HuR also regulates the levels and/or translation of other ARE-containing mRNAs which encode hypoxia-inducible proteins such as GLUT1, TGF-β, c-myc and p53 [79]. With regard to p53, HuR is able to bind Mdm2 mRNA, a critical negative regulator of p53. The authors suggest that by regulation of p53 levels HuR promotes the survival of hematopoietic progenitor cells [80]. Until now the possible role of AUF1 in those processes remains undiscovered.

Both proteins were also identified as critical mediators of replicative senescence. It had been shown that reduction of AUF1 level occurred with replicative senescence and contributed to stabilization and elevated expression of ARE-bearing p16 mRNA in senescence-phenotype cells [81]. Interestingly, exposure of the cells to hydrogen peroxide, which is related with TH production, led to reduced levels of AUF1. Furthermore, the cells overexpressing AUF1 were resistant to hydrogen peroxide-induced senescence. However whether AUF1 protective function against oxidative stress may be linked to thyroid function and disorders remains to be elucidated [82]. HuR was found to interact with the 3′UTR of the HuR mRNA and elevated HuR translation by promoting the nuclear export of HuR mRNA. The authors propose that such a regulatory mechanism may be responsible for the loss of HuR during replicative senescence [83].

Nagaoka et al. showed that AUF1 may be implicated in mammary gland differentiation. It had been demonstrated that stimulation with lactogenic hormone decreased cytoplasmic, but increased nuclear AUF1 levels. Furthermore, depletion of AUF1 correlated with elevated expression of b-casein and decreased levels of c-myc mRNAs. Such a cytoplasmic-nuclear translocation of AUF1 allowed the cells initiation of differentiation followed by induction of milk production and inhibition of proliferation [84].

## Conclusions

The possibility of functional interactions between AUF1 and HuR, and thyroid related mRNAs presented in this study is still speculative, but open new ways to investigate the unrevealed mechanisms, far beyond the known function of both proteins.

## Competing interests

The authors declare that they have no competing interests.

## List of abbreviations used

3'-UTR: 3'-untranslated region; ALK: anaplastic lymphoma kinase; ANXA2: annexin A2; ARE: Adenylate-Uridylate-rich element; AUF1: Adenylate-Uridylate rich RNA-binding Factor 1; D1, D2, D3: deiodinases I, II, III; ELAV: embryonic lethal abnormal vision; EYA1: eyes absent homolog; GAPDH): glyceraldehyde-3-phpsphate dehydrogenase; GRHL2: grainyhead-like 2; GSTP1: glutathione S-transferase P; HCV: Hepatitis C virus; HHEX: homebox protein HEX; HNRNPK: heterogeneous nuclear ribonucleoprotein K; Hox-A3: homebox protein Hox-A3; Hox-A5: homebox protein Hox-A5; hTERT: human telomerase reverse transcriptase; HuR: human antigen R; IGF2BP1: insulin-like growth factor 2 mRNA binding protein; IRES: internal ribosome entry site; MAT1A: methionine adenosyltransferase 1A; MAT1A: methionine adenosyltransferase 1A; MAT2A: methionine adenosyltransferase 2A; MAT2A: methionine adenosyltransferase 2A; MCT8: monocarboxylate transporter 8; MSH2: MutS homolog 2; NIS: sodium-iodide symporter; NPM: nucleophosmin; PGA2: Prostaglandin A2; PKM1/M2: pyruvate kinase isoenzymes M1/M2; PPIA: peptidyl-prolyl cis-trans isomerase A; PRDX2: peroxiredoxin 2; PTH: parathyroid hormone; RA: retinoic acid; RAR: retinoic acid receptors; SAM: S-adenosylmethionine; SERCA2a: sarco(endo)plasmic reticulum calcium ATPase 2a; SLE: systemic lupus erythematosus; T3: triiodothyronine; TH: thyroid hormone; THR: thyroid hormone receptor; TKT: transketolase; TPO: thyroid peroxidise; TSG: tumor suppressor genes; TSH: thyroid stimulating hormone; TSHR: thyroid stimulating hormone receptor.
